# Dissemination of Linezolid Resistance Through Sex Pheromone Plasmid Transfer in *Enterococcus faecalis*

**DOI:** 10.3389/fmicb.2020.01185

**Published:** 2020-06-04

**Authors:** Jiaqi Zou, Zhaobing Tang, Jia Yan, Hang Liu, Yingzhu Chen, Dawei Zhang, Jinxin Zhao, Yu Tang, Jing Zhang, Yun Xia

**Affiliations:** ^1^Department of Laboratory Medicine, The First Affiliated Hospital of Chongqing Medical University, Chongqing, China; ^2^Department of Urologic Surgery, The First Affiliated Hospital of Chongqing Medical University, Chongqing, China; ^3^Department of Laboratory Medicine, The Third Affiliated Hospital of Chongqing Medical University, Chongqing, China

**Keywords:** linezolid, *Enterococcus faecalis*, *optrA*, transmission, sex pheromone plasmid

## Abstract

Despite recent recognition of the ATP-binding cassette protein OptrA as an important mediator of linezolid resistance in *Enterococcus faecalis* worldwide, the mechanisms of *optrA* gene acquisition and transfer remain poorly understood. In this study, we performed comprehensive molecular and phenotypic profiling of 44 *optrA*-carrying *E. faecalis* clinical isolates with linezolid resistance. Pulse-field gel electrophoresis and DNA hybridization revealed the presence of *optrA* in the plasmid in 26 (59%) isolates and in the chromosome in 18 (41%) isolates. Conjugation experiments showed a successful transfer of *optrA* in 88.5% (23/26) of isolates carrying *optrA* in plasmids while no transfer occurred in any isolates carrying *optrA* in the chromosome (0/18). All 23 transconjugants exhibited *in vitro* resistance to linezolid and several other antibiotics and were confirmed to contain *optrA* and other resistance genes. Plasmid typing demonstrated a predominance (18/23,78%) of *rep*_9_–type plasmids (pCF10 prototype) known to be the best studied sex pheromone responsive plasmids. Full plasmid genome sequencing of one isolate revealed the presence of drug resistance genes (*optrA* and *fexA*) and multiple sex pheromone response genes in the same plasmid, which represents the first sex pheromone responsive plasmid carrying *optrA* from a clinical isolate. PCR-based genotyping revealed the presence of three key sex pheromone response genes (*prgA, prgB*, and *prgC*) in 23 *optrA*-carrying isolates. Finally, functional studies of these isolates by clumping induction assay detected different degrees of clumping in 17 isolates. Our analysis suggests that *optrA*-mediated linezolid resistance can be widely disseminated through sex pheromone plasmid transfer.

## Introduction

*Enterococcus faecalis* is an opportunistic pathogen that resides primarily in the gastrointestinal tract of most healthy individuals, but can cause urinary tract infections, surgical infections and even fatal infections, such as endocarditis and bacteremia (Arias and Murray, 2012; Beganovic et al., 2018). Its high-level intrinsic and acquired resistance to multiple drugs, especially vancomycin, has limited the treatment options (Mundy et al., 2000; Paulsen et al., 2003). There is a clear need to better understand the mechanisms of drug resistance and its transmission in order to develop effective strategies to monitor and control drug resistance.

Linezolid, the first member of oxazolidinone antibiotic antibacterial agents, was approved for clinical therapy in 2000 as the last-resort drug for treatment of serious Gram-positive bacterial infections, including vancomycin-resistant enterococci (VRE), methicillin-resistant *Staphylococcus aureus* (MRSA) and multi-drug resistant *Streptococcus pneumoniae* (Leach et al., 2011). The increasing prevalence of linezolid-resistant *E. faecalis* presents a formidable challenge to clinical treatment (Farrell et al., 2011; Deshpande et al., 2018). The common mechanisms of linezolid resistance in *E. faecalis* include point mutations in the chromosomal 23S rRNA gene or protein-coding genes encoding the L3 and L4 ribosomal proteins (Bi et al., 2018; Deshpande et al., 2018). Other newly identified mechanisms include the presence of plasmid-borne chloramphenicol-florfenicol resistance (*cfr*) gene (Schwarz et al., 2000) or ribosomal protection genes *optrA* (Wang et al., 2015) and *poxtA* (Antonelli et al., 2018).

The *optrA* gene encodes the ATP-binding cassette F (ABC-F) protein and mediates resistance to phenicols and oxazolidinones through protection of the bacterial ribosome from antibiotic inhibition (Wang et al., 2015; Sharkey et al., 2016). Following the first report of the *optrA* gene in 2015 from an *E. faecalis* isolate obtained from a blood sample of a Chinese patient (Wang et al., 2015), *optrA* has been found worldwide not only in *E. faecalis* and *E. faecium* (Cai et al., 2015; Mendes et al., 2016; Cavaco et al., 2017; Gawryszewska et al., 2017; Càmara et al., 2019; Said and Abdelmegeed, 2019; Sassi et al., 2019; Tsilipounidaki et al., 2019) but also in other Gram-positive bacteria such as *Staphylococcus sciuri* (Fan et al., 2016; Li et al., 2016) and *Streptococcus suis* (Huang et al., 2017). In addition, *optrA* has been detected not only in various human clinical samples but also in diverse livestock samples such as cow milk, and feces or meat from pigs, chicken and ducks (Elghaieb et al., 2019; Na et al., 2019) as well as environmental samples such as soils (Zhao et al., 2016) and urban wastewater (Freitas et al., 2017). Search of the PubMed database using *optrA* as the keyword in the abstract identified 2, 10, 15, 23, and 35 papers from 2015 to 2019, respectively, with the geographic origin of these reports expanded from only one country (China) in 2015 to 19 countries (from five continentals) in 2019. Such rapid, widespread dissemination of this resistance gene suggests a highly efficient dissemination capacity among animals, humans, and the environment.

Indeed, there have been reports of easy transfer of *optrA*-carrying plasmids at high transfer frequencies of 10^–2^–10^–6^ *in vitro* between *E. faecalis* and *E. faecium* (Wang et al., 2015) and between human- and pig-derived *E. faecalis* (Shang et al., 2019). Our previous transcriptomics (Hua et al., 2018) and proteomics (Yan et al., 2018) analyses of a linezolid-resistant *E. faecalis* strain P10748 consistently revealed a significant co-upregulation of *optrA* gene with several genes involved in mating and pheromone response, implying a localization of this gene in a sex pheromone plasmid allowing highly efficient resistance transfer. However, the location of the *optrA* gene and the sequences of the plasmid and chromosome of this strain have not yet been determined.

The goals of this study were to assess the distribution and transferability of the *optrA* gene in a case series of *E. faecalis* clinical isolates, determine the whole plasmid genome sequence in the linezolid-resistant *E. faecalis* strain P10748, and identify mechanisms behind *optrA* transfer. Our results suggest, for the first time, that *optrA*-mediated linezolid resistance can be widely disseminated through sex pheromone plasmid transfer.

## Materials and Methods

### Bacterial Strains and Antimicrobial Susceptibility Testing

A panel of 44 linezolid-resistant *E. faecalis* isolates was obtained from the First Affiliated Hospital of Chongqing Medical University, Chongqing, China (Hua et al., 2019). All isolates showed minimum inhibitory concentrations (MICs) > 4 mg/L of linezolid as determined by the broth microdilution method following the Clinical and Laboratory Standards Institute (CLSI, 2018) recommendations. The drug resistance phenotypes and multi-locus sequence types (MLST) of all these isolates have been determined in our previous study (Hua et al., 2019; Table 1 and Supplementary Table S1). The *E. faecalis* ATCC 29212 isolate obtained from the American Type Culture Collection (Manassas, VA, United States) served as a quality control strain. *E. faecalis* JH2-2 isolate was kindly provided by Dr. Tieli Zhou (The First Affiliated Hospital of Wenzhou Medical University, China) and used as the recipient strain in conjugation experiments.

**TABLE 1:** Characteristics of linezolid-resistance plasmids in 26 *E. faecalis* isolates carrying *optrA.*

**Isolate ID**	**Plasmid size (kb)**	**Rep-family^a^**	**Prototype^a^**	**Transferred resistance gene^a^**	**MICs of linezolid (mg/L)^b^**	**MLST^c^**
P10748	∼54	9	pCF10	*optrA, tetL, ermB, lnuB, fexA*	8	ST480
EF-3015	∼150	6, 9	pS86, pCF10	*optrA, tetL, ermB, lnuB fexA*	8	ST828
EF-7013	∼60	9	pCF10	*optrA, tetL, ermB, lnuB, fexA*	8	ST16
EF-8194	∼80	9	pCF10	*optrA, tetL ermB, lnuB fexA*	8	ST16
EF-9289	∼80	1, 7, 9	pIP501, pUSA02, pAD1	*optrA, tetL, ermB, fexA*	8	ST826
EF-3139	∼100	1, 9	pIP501, pCF10	*optrA, tetL, tetM, ermB, lnuB fexA*	16	ST386
EF-5136	∼80	9	pCF10	*optrA, tetL, tetM, ermB, lnuB fexA*	8	ST16
EF-2084	∼80	1, 9	pIP501, pCF10	*optrA, tetL, tetM, ermB, lnuB, fexA*	8	ST480
EF-0132	∼80	1, 9	pIP501, pCF10	*optrA, tetL ermB, lnuB, fexA*	16	ST585
EF-4003	∼55, ∼350^d^	7	pUSA02	*optrA, tetL, tetM, ermB, fexA*	16	ST16
EF-8042	∼30, ∼200^d^	1, 9	pIP501, pCF10	*optrA, tetL, ermB, fexA*	8	ST69
EF-0074	∼65	7, 9	pUSA02,pAD1	*optrA, tetL, ermB, lnuB, fexA*	8	ST826
EF-6165	∼80	1	pIP501	*optrA, tetL, tetM, ermB, lnuB, fexA*	8	ST823
EF-7094	∼40, ∼350^d^	9	pCF10	*optrA, tetL, tetM, ermB, lnuB, fexA*	8	ST632
EF-1127	∼55	1, 9	pIP501, pCF10	*optrA, tetL, tetM, ermB, lnuB, fexA*	8	ST824
EF-8137	∼80	1, 7, 9	pIP501, pUSA02, pCF10	*optrA, tetL, tetM, ermB, lnuB, fexA*	8	ST116
EF-8282	∼80	6,9	pS86, pCF10	*optrA, tetL, ermB, lnuB*	16	ST330
EF-4245	∼80	9	pCF10	*optrA, tetL, ermB, lnuB, fexA*	8	ST823
EF-1080	∼40	9	pCF10	*optrA, tetL, tetM, ermB, lnuB, fexA*	8	ST69
EF-2216	∼80	1,9	pIP501,pCF10	*optrA, tetL, tetM, ermB, lnuB, fexA*	8	ST376
EF-6166	∼80	NA	NA	*optrA, tetL, ermB, lnuB, fexA*	16	ST16
EF-5015	∼80	NA	NA	*optrA, tetL, tetM, ermB, lnuB, fexA*	8	ST480
EF-2021	∼90	NA	NA	*optrA, tetL, ermB, lnuB fexA*	16	ST714
EF-8014	∼55	NT	NT	NT	8	ST632
EF-3186	∼30	NT	NT	NT	8	ST69
EF-1090	∼55	NT	NT	NT	8	ST585

*^*a*^These results are consistent between transconjugants and original clinical isolates. ^*b*^All clinical isolates considered low-level resistance to linezolid (Hua et al., 2019). MIC values for other drugs are listed in Supplementary Table S1. ^*c*^Multi-locus sequence types determined for original isolates in our previous study (Hua et al., 2019). ^*d*^Two plasmids with different sizes. NA, not available in plasmid typing with 19 known *rep* families. NT, not tested due to negative results in conjugation experiment.*

### Determination of the *optrA* Location

To determine the location of *optrA*, S1 nuclease-pulsed field electrophoresis (S1-PFGE) and Southern blot were performed following the methods described previously (Barton et al., 1995; Rosvoll et al., 2010). Briefly, bacterial cells harvested from fresh culture were embedded in agarose gels and then digested by S1 nuclease (TaKaRa, Dalian, China). *Salmonella Braenderup* H9812 chromosomal DNA was digested with *Xba*I (TaKaRa, Dalian, China) and used as the DNA size marker. CHEF electrophoresis was performed using the same conditions described elsewhere (Rosvoll et al., 2010). Following transfer to Hybond N + membranes (Amersham Biosciences, United States) by capillary blotting for more than 20 h, blots were hybridized with digoxigenin (DIG)-labeled *optrA*-specific probe using the DIG High Prime DNA Labeling and Detection Starter Kit II (Roche Applied Sciences, Germany) following the manufacturer’s instructions.

### Conjugation Experiments and Detection of Resistance Genes

To examine the transferability of plasmids containing *optrA*, filter mating was conducted on nitrocellulose filters (0.45 μm pore size, Millipore, United States) as described previously (Werner et al., 2003). Rifampicin-resistant *E. faecalis* JH2-2 was used as a recipient strain and *optrA*-positive *E. faecalis* was used as a donor strain. The donor and recipient strains were cultured to the exponential growth phase (OD_600_ = 0.4–0.6) and then mixed at a ratio of 1:3. The mixture was shaken for 30 min and centrifuged. The pellet was placed on a filter for overnight incubation at 37°C. Transconjugants were selected on Brain Heart Infusion (BHI) agar (Solarbio, Beijing, China) supplemented with 25 mg/L of rifampin and 10 mg/L of florfenicol. Subsequently, the selected transconjugants were subjected to confirmation of the presence or absence of the *optrA* gene and the resistance to rifampicin and florfenicol. The use of florfenicol instead of linezolid for selecting transconjugants was based on the observation of a larger difference for florfenicol than for linezolid in MIC values (4 vs. 64 mg/L) between the donor and recipient bacteria. Approximately 20–150 colonies were obtained from each transconjugation reaction, with 2–5 of them selected for PCR analysis.

To detect the transmission of resistance in transconjugants, antimicrobial susceptibility of the transconjugants were determined as described above. The presence of known potential antibiotic resistance genes including *tetM*, *tetL*, *ermB, lnuB* and *fexA*, were screened by PCR amplification using the primers listed in Supplementary Table S2. Positive PCR products were sequenced commercially by Sangon Biotech (Shanghai, China). Resulting sequences were analyzed using MegAlign (version 7.1.0, DNASTAR, United States) and compared with reference sequences in the NCBI nucleotide database.

### Plasmid Replicon Typing

On the basis of plasmid classification schemes by Jensen et al. (2010), we designed PCR primers to detect 19 different types of *rep*-family plasmids (*rep*_1__–__11_, *rep*_13__–__19_ and *rep*_unique_) in *E. faecalis* transconjugants. *Rep*_12_ family was not included owing to lacking a reference strain. All samples were first screened by multiplex PCR, followed by single-locus PCR for samples that were not clearly differentiated in multiplex PCR. All positive PCR products were sequenced commercially and analyzed against sequences of known *rep*-families in the NCBI nucleotide database. In addition to transconjugants, the *rep*-types in all original isolates were also assessed by the same PCR method.

### Sequencing and Analysis of the Whole Plasmid Genome in P10748 Harboring *optrA*

The whole plasmid genome in the *E. faecalis* strain P10748 was sequenced as a part of the *E. faecalis* genome sequencing project [U.S. National Center for Biotechnology Information (NCBI, Bethesda, MD, United States) BioProject accession no. PRJNA629062]. Genomic DNA was extracted from overnight culture using the Qiagen Plasmid Midi Kit (Qiagen, Germany) according to the manufacturer’s instructions. Five micrograms of DNA were used to construct one Illumina library and sequenced using 250 base paired-end reads on the Illumina HiSeq platform commercially (Novogene Corporation, Hong Kong). A total of 20 million reads were obtained. After removal of low-quality reads and reads for *E. faecalis* nuclear genome sequences (GenBank accession no. CP008816), the remaining 285,862 reads were assembled using SeqMan NGen (version 14.1.0.118, DNASTAR, Madison, WI, United States), resulting in a circular assembly of the complete plasmid genome. This assembly was validated by PCR and Sanger sequencing of multiple overlapping fragments, and by restriction mapping using *Nhe*I.

Gene prediction was conducted through a combination of multiple computational programs including GeneMarkS (Besemer et al., 2001), RAST (Aziz et al., 2008), ORF Finder^1^ and BLAST^2^.

### Distribution of Key Sex-Pheromone-Response Genes in *optrA*-Carrying *E. faecalis* Isolates and Their Transconjugants

Genomic DNA was extracted from overnight cultures using the HiPure Bacterial DNA Kit (Magen, Guangzhou, China) according to the manufacturer’s protocol. The presence of three key sex-pheromone-response genes (*prgA, prgB*, and *prgC*) in the donor and transconjugant strains was determined by PCR using primers listed in Supplementary Table S2. All positive PCR products were sequenced and the resulting sequences were blasted against the NCBI nucleotide database.

### Determination of Sex Pheromone Responses by Clumping Induction Assay (CIA)

Clumping induction assay (CIA) was performed as described previously (Donelli et al., 2004). Pheromone-containing filtrates were prepared from *E. faecalis* JH2-2 strain grown overnight in BHI broth (referred to as the recipient strain). The cells were pelleted by centrifugation, and the supernatant was filtered through a 0.22 μm filter membrane. The filtrates were autoclaved at 121°C for 20 min, and stored at 4°C before use. *E. faecalis* culture (from clinical isolates or their transconjugants, referred to as the donor strain) was added to pheromone-containing filtrate, followed by incubation at 37°C for 3 h with shaking. To test the type of sex pheromones involved in mating, synthetic sex pheromone cAD1 or cCF10 (Sangon Biotech, Shanghai, China) was used at the final concentration of 100 ng/ml instead of JH2-2-derived filtrates described above (Lim et al., 2006; Zheng et al., 2009). Degrees of cell clumping were semi-quantitated as titers for visible cell aggregates.

### Quantification of Transfer Frequencies for Plasmid pEF10748 by Short Mating Assay

Short mating induced by the pheromone was performed as described previously (Lim et al., 2006). After 45-min pheromone induction, 0.1 ml of the donor strain (P10748) was mixed with 0.9 ml of the recipient strain *E. faecalis* JH2-2, followed by incubation for 15 min at 37°C. The mixture was then plated on selective plates containing 25 mg/L of rifampin and 10 mg/L of florfenicol.

## Results

### Localization of *optrA* Gene in the Linezolid-Resistant *E. faecalis*

**FIGURE 1:**
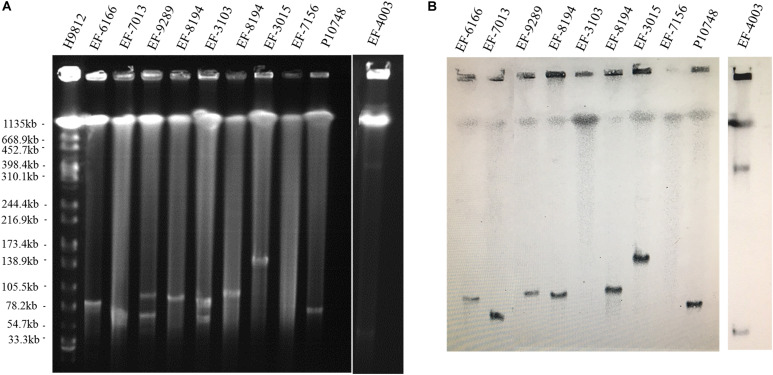
Determination of the location of *optrA* in linezolid-resistant *Enterococcus faecalis* isolates. **(A)** Representative results of S1 nuclease-pulsed-field gel electrophoresis analysis. The first lane contained *Xba*I-digested chromosome of *Salmonella Braenderup* H9812 as a DNA size marker. **(B)** Representative results of Southern hybridization showing the location of *optrA* in plasmids or chromosomes. IDs of individual isolates analyzed are indicated at the top.

Among 44 linezolid-resistant *E. faecalis* isolates analyzed by S1-PFGE, *optrA* was localized on the plasmid in 26 (59%) isolates and on the chromosome in 18 (41%) isolates. The size of plasmids varied from 30 to 300 kb (Figure 1 and Supplementary Figure S1). Of the 26 isolates with *optrA*-carrying plasmids, 23 had a single plasmid and the remaining 3 had a co-existence of two plasmids with different sizes (Table 1).

### Horizontal Transmission of *optrA* Gene

In conjugation experiments with *E. faecalis* JH2-2 as a recipient, positive transfer of the *optrA* gene was achieved in 23/26 (88.5%) *E. faecalis* clinical isolates with *optrA*-carrying plasmids. The transfer frequencies varied from 10^–3^ to 10^–6^ per donor cell. The other three isolates showed no transfer despite multiple attempts. None of the 18 isolates containing *optrA* in the chromosome showed positive transfer. In drug susceptibility testing of the 23 transconjugants, the MIC value of linezolid was 8–16 mg/L whereas the recipient strain was 2 mg/L. In addition, these 23 transconjugants exhibited resistance to chloramphenicol, clindamycin, erythromycin, and tetracycline compared with the recipient strain *E. faecalis* JH2-2 (data not shown). By PCR, in each of the 23 transconjugants we detected multiple resistance genes, including *optrA* (23/23, 100%), *tetL* (23/23, 100%), *tetM* (11/23, 47.8%), *ermB* (22/23, 95.6%), *lnuB* (20/23, 87.0%), and *fexA* (22/23, 95.6%) (Table 1). The resistance gene profiles of transconjugants were consistent with those of the donor isolates.

### Detection of *rep*-Families in *E. faecalis* Transconjugants and Their Original Clinical Isolates

Four types of plasmids were identified by PCR in 20 isolates carrying *optrA* while other three isolates did not show any of the 19 plasmid types targeted in our study (Table 1). The dominant plasmid type was *rep*_9_ family (prototype pCF10), which was detected in 78% (18/23) of isolates. *Rep*_1_ (prototype pIP501) accounted for the second major *rep*-family being identified in 39% (9/23) isolates. *rep*_7_ (pIP501 prototype) and *rep*_6_ (pIP501 prototype) were detected with lower frequencies (17.4% or 4/23 and 8.7% or 2/23, respectively). Other plasmids including *rep*_2__–__5_ *rep*_8_, *rep*_10__–__11_, *rep*_13__–__16_, *rep*_18__–__19_, and the *rep*_*Unique*_ were not detected in any isolates.

The results of *rep* types were consistent between transconjugants and their corresponding original isolates. Of the 20 successfully typed isolates, 9 contained a single plasmid type, 9 contained a mixture of two plasmid types and the remaining 2 contained a mixture of three plasmid types (Table 1).

### Distribution of Key Sex-Pheromone-Response Genes in *optrA*-Carrying *E. faecalis* Isolates and Their Transconjugants

Among the 23 original isolates and their respective transconjugants carrying *optrA*, 15 were PCR-positive for the *prgA* gene with both the original isolates and their transconjugants, 5 were positive with the original isolate only, and the remaining 3 were negative (Table 2). In PCR testing for the *prgB* gene, 21 were positive with both the original isolates and transconjugants, and the remaining 2 were positive with the original isolate only. In PCR testing for the *prgC* gene, 10 were positive with both the original isolates and transconjugants, 5 were positive with the original isolate only, and the remaining 8 were negative with both the original isolates and transconjugants. Overall, all 23 isolates were positive for at least one of the three key pheromone-response genes with either the original isolates or transconjugants; only seven isolates were positive for all three genes with both the original isolates and transconjugants. *prgB* was the most common gene, which was detected in all 23 original isolates.

**TABLE 2:** Distribution of key sex-pheromone-response genes and results of clumping assay in 23 *E. faecalis* isolates showing successful transfer of *optrA*-carrying plasmids.

	**Pheromone-response**						
	**genes^a^**				**Clumping assay^b^**		
	***rep9***	***prgA***	***prgB***	***prgC***	**cCF10**	**cAD1**	**JH2-2 filtrate**
P10748	+/+	+/+	+/+	+/+	8	2	2
EF-3015	+/+	−/+	+/+	−/−	2	16	2
EF-7013	+/+	−/+	+/+	+/+	32	32	8
EF-8194	+/+	+/+	+/+	+/+	64	32	4
EF-9289	+/+	+/+	+/+	−/−	8	16	4
EF-3139	+/+	+/+	+/+	+/+	−	2	2
EF-5136	+/+	+/+	+/+	−/+	−	−	2
EF-2084	+/+	−/+	+/+	−/−	−	−	2
EF-0132	+/+	+/+	+/+	+/+	2	−	4
EF-4003	−/−	+/+	+/+	−/−	−	−	−
EF-8042	+/+	+/+	+/+	+/+	−	−	2
EF-0074	+/+	+/+	+/+	+/+	32	64	4
EF-6165	−/−	+/+	+/+	+/+	−	−	−
EF-7094	+/+	−/+	+/+	+/+	−	−	2
EF-1127	+/+	−/−	+/+	−/−	−	−	2
EF-8137	+/+	+/+	+/+	−/+	2	2	4
EF-8282	+/+	+/+	+/+	−/+	2	−	2
EF-4245	+/+	−/−	+/+	−/+	−	−	2
EF-1080	+/+	−/−	+/+	−/−	2	−	2
EF-2216	+/+	−/+	−/+	−/+	−	−	−
EF-6166	−/−	+/+	+/+	−/−	−	−	−
EF-5015	−/−	+/+	−/+	+/+	−	−	−
EF-2021	−/−	+/+	+/+	−/−	−	−	−

*^*a*^The presence (+) or absence (−) in transconjugants/original clinical isolates by PCR. ^*b*^Titers for visible cell aggregates with original isolates only. The values represent dilution factors from stock concentration, eg., 8 represents 1:8.*

Of the 18 original isolates containing a *rep*_9_ type of plasmids, all were positive for *prgB*, 15 were positive for *prgA* and 13 were positive for *prgC*. On the other hand, of the 5 original isolates that did not carry a *rep*_9_ type of plasmids, all were positive for *prgA* and *prgB* while only 2 were positive for *prgC*.

### Determination of Sex Pheromone Responses by CIA

In CIA assay with synthetic pheromones cAD1 and cCF10 as inducers, 11 of 23 *optrA*-carrying isolates showed different degrees of clumping with at least one inducer, including three showing titers higher than 1:16 and 9 showing titers from 1:2 to 1:16 (Table 2). None of the 5 isolates without a *rep*_9_-type plasmid showed apparent clumping. In CIA assay with JH2-2 filtrates, 17 of 23 *optrA*-carrying isolates (all containing *rep*_9_-type plasmids) displayed aggregation with titers from 1:2 to 1:8 while the remaining 6 isolates showed no aggregation.

### Quantification of Transfer Frequencies of the Plasmid pEF10748 by Short Mating

Transfer frequencies were determined by short mating assay using induced or uninduced donor *E. faecalis* containing plasmid pEF10748 and plasmid-free recipient *E. faecalis* JH2-2 (Table 3). With induced donor cells, the transfer frequency was about 10^–4^ per donor cell while with uninduced donor cells, the transfer frequency was about 10^–8^ per donor cell, supporting that the plasmid pEF10748 conferred a pheromone response. Compared to uninduced cells, pEF10748 responded to both cCF10 and cAD1 with higher transfer frequencies (∼10^–6^), which suggests that pEF10748 functions similarly to the pheromone-responsive plasmids pCF10 and pAD1 (Dunny et al., 1982; Clewell, 2007).

**TABLE 3:** Plasmid transfer frequency in *E. faecalis* isolate P10748 carrying sex pheromone plasmid pEF10748 based on short mating assay.

**Donor**	**Recipient**	**Exposure to pheromone**	**Transfer frequency^a^**
			
P10748	JH2-2	-	2 × 10^–8^
		JH2-2 filtrate	4 × 10^–4^
		cCF10	3 × 10^–6^
		cAD1	1.2 × 10^–6^

*^*a*^No. of transconjugants per donor cell.*

### Characteristics of the *optrA-Carrying* Plasmid in *E. faecalis* Strain P10748

**FIGURE 2:**
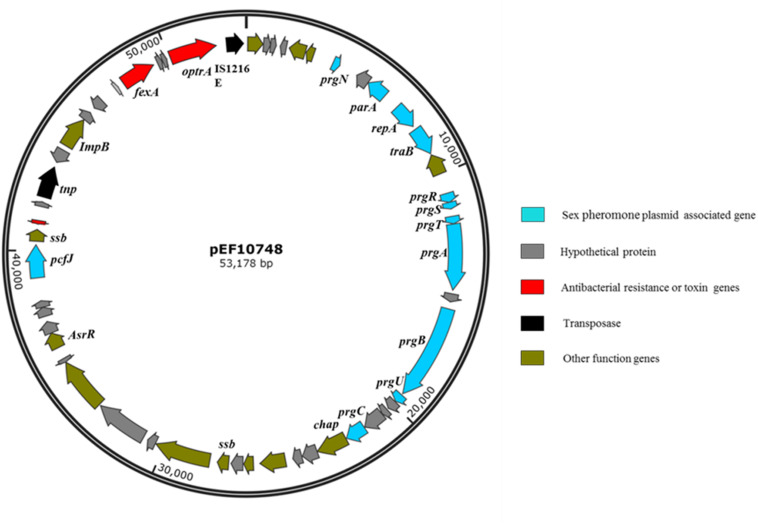
Gene organization in plasmid pEF10748 from the *E. faecalis* clinical isolate P10748. Arrows indicate the CDSs and their transcription directions. The putative functions of CDSs are color-coded as indicated at the right.

*De novo* assembly of Illumina HiSeq data from *E. faecalis* P10748 resulted in a complete genome of plasmid pEF10748 containing 53,178 bp (GenBank accession no. MK993385.1). This size is consistent with the results of Southern blot (Figure 1), supporting the correct assembly of the plasmid genome. This genome has a GC content of 35%, similar to that of the *E. faecalis* genome. A total of 58 coding sequences (CDSs) were identified, with the majority of them encoded in the same orientation, similar to known pheromone plasmids in *E. faecalis*, such as pCF10 (Hirt et al., 2005) and pAD1 (Clewell, 2007). The predicted genes of all CDSs are listed in Supplementary Table S3 and Figure 2.

**FIGURE 3:**
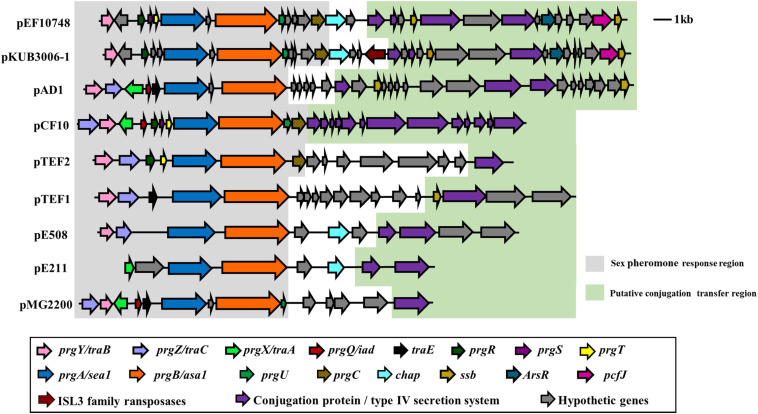
Comparison of the sex pheromone gene cluster among known sex pheromone plasmids. Different genes are color-coded as shown in the box on the bottom. Of note, for the region shown, the gene organization in plasmid pEF10748 identified in this study is most similar to that of pKUB3006-1 reported by Kuroda et al. (2018). The bacterial origin and GenBank accession numbers of all plasmids shown are available from Supplementary Table S4.

The full-length sequence of plasmid pEF10748 was not identical to any plasmid sequences currently available in GenBank while being most closely related to plasmid pKUB3006-1 in an *E. faecalis* isolate reported from Japan (Kuroda et al., 2018), with 78% coverage and 99% sequence identity for a ∼42 kb region (Supplementary Table S4). Other plasmids with significant overlap to this region included pTEF1, pAD1, pMG2200, pCF10 and pTEF2 (Figure 3). All these plasmids were identified in *E. faecalis* from human and swine samples and contain multiple sex pheromone response genes, including *traB*, *prgA* (*sea1*), *prgB* (*asa1*), *prgC, prgU*, *prgR*, *prgS*, and *prgT.*

**FIGURE 4:**
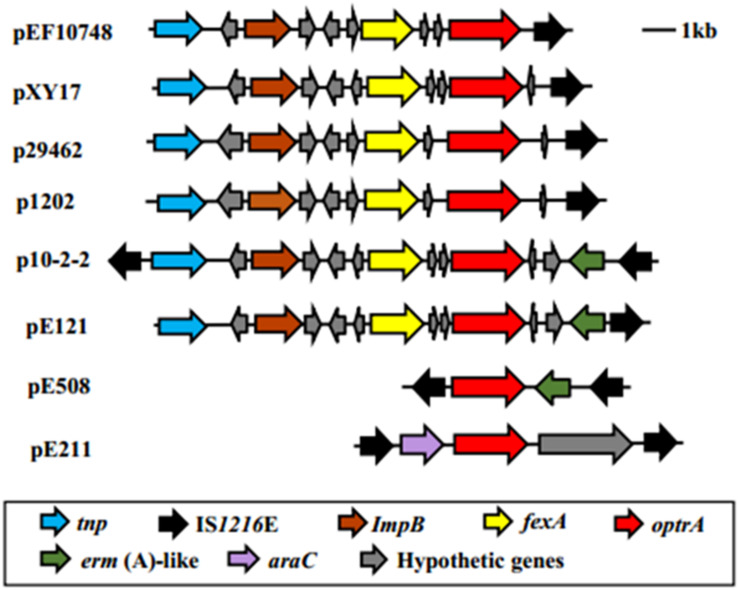
Comparison of the genetic environment of the linezolid resistance *optrA* among different plasmids. Different genes are color-coded as shown in the box on the bottom. Of note, for the region shown, the gene organization in plasmid pEF10748 identified in this study is most similar to that of pXY17 reported by He et al. (2016). The bacterial origin and GenBank accession numbers of all plasmids shown are available from Supplementary Table S4.

The *optrA* gene in plasmid pEF10748 was surrounded by chloramphenicol/florfenicol resistance gene *fexA*, ISL3 transposase, *Imp* and IS1216E element, all of which were located in a ∼11 kb region (Supplementary Table S3 and Figure 2). This region showed highest homology (>99.9% sequence identity) to the following plasmids: pXY17, p10-2-2, p29462, p1202, and pE121 (Supplementary Table S4 and Figure 4). All these plasmids were identified from *E. faecalis* in China.

## Discussion

In this study, we performed comprehensive molecular and phenotypic profiling of 44 *E. faecalis* clinical isolates with linezolid resistance through combination of PFGE, DNA hybridization, PCR-based genotyping, whole plasmid genome sequencing, antibiotic susceptibility testing, bacterial conjugation and clumping induction assay. Our results strongly suggest that *optrA*-mediated linezolid resistance can be widely disseminated through sex pheromone plasmid transfer.

Our conclusion on the sex pheromone plasmid transfer mechanism is supported by the following findings.

First, we detected a high prevalence of plasmid-borne *optrA* gene in *E. faecalis* clinical isolates. The proportion of isolates carrying *optrA* in the plasmid was slightly higher than isolates carrying *optrA* in the chromosome (59 vs. 41%), which is consistent with previous reports in China (Cai et al., 2015; Wang et al., 2015) and other countries (Bender et al., 2018; Deshpande et al., 2018; Sassi et al., 2019). It is well-known that plasmids can mediate fast and efficient gene transfer within and between different bacterial host species.

Second, we detected a high transferability (23/26, 88%) of *optrA*-carrying plasmids based on studying a case series of 26 *E. faecalis* clinical isolates with linezolid resistance and carrying *optrA* in plasmids (Table 1). This transferability was confirmed by MIC testing and PCR amplification of multiple genes (drug-resistance genes, rep genes and sex-pheromone-response genes) in all transconjugants. In addition, quantitative study of the isolate *E. faecalis* P10748 carrying an *optrA*-containing plasmid revealed a high plasmid transfer frequency (10^–4^). To our knowledge, this study is the largest to date to assess the transferability of *optrA*-carrying plasmids in clinical isolates of *E. faecalis.* Based on the results of drug resistance profiles (Table 1), plasmid replicon typing and distribution of key sex-pheromone-response genes (Table 2), more than a half (13 or 57%) of the 23 transconjugants showed identical patterns as the original isolates, suggesting the likelihood of transferring the same plasmid, while the remaining transconjugants (10 or 43%) showed differences in some properties (e.g., the distribution of 3 key sex-pheromone-response genes) (Table 2), suggesting the likelihood of transferring different genetic materials. Thorough confirmation of the exact transferred materials will require additional experiments such as whole chromosome and plasmid sequencing, and high-resolution PFGE coupled with probe hybridization.

Third, sequencing of the full plasmid genome in one *E. faecalis* clinical strain (P10748) confirmed the co-localization of *optrA* with almost all known sex-pheromone response genes, including the typical *prgA-prgB-prgC* cassette, in the same plasmid (Figure 2). Plasmids with such gene organization have not been reported previously from any *optrA*-carrying *E. faecalis* isolates. While there has been one report of two pheromone-responsive plasmids carrying *optrA* in *E. faecalis* (Shang et al., 2019), both plasmids were identified in *E. faecalis* isolates from pigs, with only one of them (pE508) containing a single sex pheromone gene (*prgB*) based on full plasmid genome sequencing.

Fourth, our plasmid typing studies demonstrated, for the first time, the abundant presence of *rep*_9_-type plasmids (prototype pCF10) in *optrA*-carrying *E. faecalis* clinical isolates (17/26, 65%, Table 1), implying a high prevalence of sex pheromone plasmids in clinical isolates. Consistent with this observation, our PCR-based genotyping revealed the presence of three key sex pheromone response genes (*prgA, prgB*, and *prgC*) in almost all *optrA*-carrying *E. faecalis* clinical isolates (Table 2). It is noteworthy that multiple *rep* types were detected in 10 *E. faecalis* isolates which showed a single plasmid in PFGE; this could be explained either by the failure in separating multiple plasmids with similar sizes due to low resolution of the PFGE gel, or by the presence of a hybrid plasmid containing multiple *rep* types as has been reported previously (Pavlova et al., 2002). It is also noteworthy that a single *rep* type was detected in two *E. faecalis* isolates which showed two plasmids with different sizes; this could be explained either by a potential partial degradation of the plasmid in the process of PFGE, or by the presence of two plasmids which contained the same rep gene family and may differ in other coding and/or non-coding regions. Clarification of these possibilities requires further investigation by other approaches particularly the long-read next-generation sequencing.

Fifth, our functional studies with CIA (induced by JH2-2 filtrate) detected different degrees of cell clumping in most of the 23 *E. faecalis* clinical isolates (17/23, 73.9%) carrying *optrA* in plasmids, indicating that pheromone-inducible conjugation is operational in these isolates. The absence of clumping in some of these isolates may be caused by the presence of other pheromone receptors different from those for cAD1 and cCF10 or JH2-2 infiltrate used in the CIA assay.

Finally, the hypothesis of sex pheromone-mediated transfer of *optrA* is further supported by our previous transcriptomics (Hua et al., 2018) and proteomics (Yan et al., 2018) studies of the *E. faecalis* P10748, which consistently showed that OptrA and several sex pheromone response molecules (PrgA, PrgB, and PrgY) were among the most significantly up-regulated molecules.

The plasmid pEF10748 identified in this study represents a novel plasmid for *E. faecalis*. In addition to the presence of *optrA* and multiple sex pheromone response genes described above, this plasmid contains other drug resistance and virulence determinants. The *fexA* gene, which confers resistance to chloramphenicol and florfenicol, is located closely to the *optrA* gene in pEF10748. The region containing these two drug resistance genes is flanked by two transposase genes including the IS1216 and ISL3 family transposases (Figure 2). The same gene organization has been reported in only one partially sequenced plasmid (pXY17) from an *E. faecalis* clinical isolate (He et al., 2016). It is likely that this organization will facilitate the movement of the resistance genes to different locations (plasmids or chromosomes) or different bacteria. This possibility awaits further investigation. In addition to drug resistance genes, CDS45 in plasmid pEF10748 encodes a putative holin-like toxin (Supplementary Table S3), which is known to function through disruption of the host cell membrane (Saier and Reddy, 2015). Whether it performs a similar function in *E. faecalis* awaits future investigation. Of note, a homolog to the pheromone receptor prgZ/traC was not identified in the plasmid PEF10748 despite rigorous search with different approaches. This absence has also been reported for pheromone-responsive plasmid pE211 in *E. faecalis* E211 (Shang et al., 2019). It is unknown how this absence affects the transfer of the plasmid pEF10748. We suspect that this absence may reduce the efficiency of plasmid conjugation, given that the transfer frequencies we observed with this plasmid appeared to much lower compared to pheromone-responsive plasmids containing prgZ/traC (Dunny et al., 1982; Lim et al., 2006) and mutant strains lacking PrgZ can still respond to the pheromone (Leonard et al., 1996).

Our finding of the integration of the *optrA* resistance gene into a pheromone-responsive plasmid may explain, at least partially, the increasing, wide dissemination of linezolid resistance all over the world. According to surveillance of antibiotic resistance in 2019 from, China 63 hospitals in Chongqing (unpublished data), up to 2.1% (60/2871) of *E. faecalis* clinical isolates have developed resistance to linezolid while no vancomycin resistance (0/2948) was detected. Although there have been reports of successful *in vitro* transfer of *optrA*-carrying plasmids from *E. faecalis* into vancomycin-resistant *E. faecium* (Wang et al., 2015), there is no report to date of clinical isolates of *Enterococcus* sp. showing resistance to both linezolid and vancomycin in China. Vancomycin resistance gene has also been found on pheromone-responsive plasmids (Magi et al., 2003; Lim et al., 2006; Zheng et al., 2009) though the mechanism of how the resistance is regulated by the plasmid remains unclear.

This study clearly has some limitations. The distribution of key sex pheromone response genes in clinical isolates was determined by PCR alone using total genomic DNA, which is unable to distinguish whether these genes are located in plasmids. Clarification of this question requires further studies by DNA hybridization or full plasmid genome sequencing. Despite the availability of a relative large collection of *optrA*-carrying plasmids, we sequenced only one of them (strain P10748), which was chosen due to our previous studies by transcriptome (Hua et al., 2018) and proteome (Yan et al., 2018) analysis. It is unknown if the remaining plasmids also carry sex pheromone response genes as well as other mobile genetic elements, why some isolates showed a single plasmid but multiple *rep* types, and why some isolates showed multiple plasmids but a single *rep* type. We anticipate that continuing decrease in next-generation sequencing cost should allow us to sequence all these plasmids as well as related chromosomes, and provide answers to these questions in the future.

## Conclusion

This report represents the largest study of the prevalence and transferability of *optrA*-carrying plasmids in *E. faecalis* clinical isolates, and the first identification of a plasmid carrying *optrA* along with multiple sex pheromone response genes in clinical isolates. Our integrative molecular and phenotypic analysis strongly suggests that *optrA*-mediated linezolid resistance can be widely disseminated through sex pheromone plasmid transfer. Further studies are needed to determine how this transfer is regulated and what the best strategy to monitor and control the transmission is.

## Author’s Note

This manuscript has been released as a pre-print at BioRxiv (Zou et al., 2019).

## Data Availabilty Statement

The dataset for this study can be found in the GenBank: https://www.ncbi.nlm.nih.gov/nuccore/MK993385).

## Author Contributions

YX designed the study. JZou and ZT collected the data and performed experiments. JY, HL, YC, and JZhao assisted in experiments. DZ, JZhang, and YT analyzed the data. All authors contributed to data drafting, revising, and approved the final submission.

## Conflict of Interest

The authors declare that the research was conducted in the absence of any commercial or financial relationships that could be construed as a potential conflict of interest.

## Footnotes

1 https://www.ncbi.nlm.nih.gov/orffinder/

2 http://blast.ncbi.nlm.nih.gov/Blast.cgi
