# Supplementary material for: Dissemination of Linezolid Resistance Through Sex Pheromone Plasmid Transfer in Enterococcus faecalis
Source: Front Microbiol. 2020 Jun 4;11:1185. doi: 10.3389/fmicb.2020.01185 (PMC7288747; doi:10.3389/fmicb.2020.01185)
Supplement: Supplementary file 1 [file Data_Sheet_1.docx]

**Supplementary Table S1**. Antimicrobial susceptibility of 26 clinical *E. faecalis* isolates carrying *optrA* determined by VITEK 2

| Isolate ID | Minimum inhibitory concentration (mg/L)^a^ | | | | | | | | | | | | | | |
| --- | --- | --- | --- | --- | --- | --- | --- | --- | --- | --- | --- | --- | --- | --- | --- |
|  | AMP | PEN | CIP | MOX | LEV | TET | ERY | HLSR | HLGR | CLI | VAN | TIG | DAF | FUR | LZD |
| P10748 | ≤2，S | 1，S | 2，I | 0.5，S | 4，I | ≥16，R | ≥8，R | SYN-R | SYN-R | ≥8，R | 1，S | ≤0.12，S | 2，R | ≤16，S | 8 |
| EF-3015 | ≤2，S | 1，S | 2，I | 0.5，S | 4，I | ≥16，R | ≥8，R | SYN-R | SYN-R | ≥8，R | 1，S | ≤0.12，S | 2，R | ≤16，S | 8 |
| EF-7013 | ≤2，S | 2，S | ≥8，R | ≥8，R | ≥8，R | ≥16，R | ≥8，R | SYN-R | SYN-R | ≥8，R | 1，S | ≤0.12，S | 8，R | ≤16，S | 8 |
| EF-8194 | ≤2，S | 2，S | ≥8，R | ≥8，R | ≥8，R | ≥16，R | ≥8，R | SYN-R | SYN-S | ≥8，R | 1，S | ≤0.12，S | 4，R | ≤16，S | 8 |
| EF-9289 | ≤2，S | 8，R | 2，I | 0.5，S | 2，S | ≥16，R | ≥8，R | SYN-S | SYN-R | ≥8，R | 2，S | ≤0.12，S | 8，R | ≤16，S | 8 |
| EF-3139 | ≤2，S | 8，R | 1，S | 0.5，S | 1，S | ≥16，R | ≥8，R | SYN-R | SYN-R | ≥8，R | 1，S | ≤0.12，S | 8，R | ≤16，S | 16 |
| EF-5136 | ≤2，S | 2，S | 2，I | 0.5，S | 2，S | ≥16，R | ≥8，R | SYN-S | SYN-S | ≥8，R | 1，S | ≤0.12，S | 4，R | ≤16，S | 8 |
| EF-2084 | ≤2，S | 2，S | ≥8，R | ≥8，R | ≥8，R | ≥16，R | ≥8，R | SYN-R | SYN-R | ≥8，R | 1，S | ≤0.12，S | 8，R | 32，S | 8 |
| EF-0132 | ≤2，S | 2，S | ≥8，R | ≥8，R | ≥8，R | ≥16，R | ≥8，R | SYN-R | SYN-R | ≥8，R | 1，S | ≤0.12，S | 4，R | ≤16，S | 16 |
| EF-4003 | ≤2，S | 4，S | ≥8，R | ≥8，R | ≥8，R | ≥16，R | ≥8，R | SYN-R | SYN-R | ≥8，R | 1，S | ≤0.12，S | 8，R | ≤16，S | 16 |
| EF-8042 | ≤2，S | 1，S | ≥8，R | 4，R | ≥8，R | ≥16，R | ≥8，R | SYN-S | SYN-S | ≥8，R | 1，S | ≤0.12，S | 8，R | ≤16，S | 8 |
| EF-0074 | ≤2，S | 2，S | ≥8，R | ≥8，R | ≥8，R | ≥16，R | ≥8，R | SYN-R | SYN-R | ≥8，R | 1，S | ≤0.12，S | ≥16，R | ≤16，S | 8 |
| EF-6165 | ≤2，S | 8，R | ≥8，R | 2，I | ≥8，R | ≥16，R | ≥8，R | SYN-R | SYN-S | ≥8，R | 1，S | ≤0.12，S | 0.5，R | 128，R | 8 |
| EF-7094 | ≤2，S | 2，S | 2，I | 0.5，S | 4，I | ≥16，R | ≥8，R | SYN-S | SYN-R | ≥8，R | 1，S | ≤0.12，S | ≥16，R | ≤16，S | 8 |
| EF-1127 | ≤2，S | 2，S | 2，I | 0.5，S | 4，I | ≥16，R | ≥8，R | SYN-R | SYN-R | ≥8，R | 1，S | ≤0.12，S | ≥16，R | ≤16，S | 8 |
| EF-8137 | ≤2，S | 2，S | 4，R | 1，S | 2，S | ≥16，R | ≥8，R | SYN-R | SYN-S | ≥8，R | 1，S | ≤0.12，S | 8，R | ≤16，S | 8 |
| EF-8282 | ≤2，S | 2，S | ≥8，R | 2，I | ≥8，R | ≥16，R | ≥8，R | SYN-R | SYN-S | ≥8，R | 1，S | ≤0.12，S | 4，R | ≤16，S | 16 |
| EF-4245 | ≤2，S | 8，R | ≥8，R | 1，S | ≥8，R | ≥16，R | ≥8，R | SYN-R | SYN-S | ≥8，R | 1，S | ≤0.12，S | 0.5，R | ≤16，S | 8 |
| EF-1080 | ≤2，S | 2，S | ≥8，R | 4，R | ≥8，R | ≥16，R | ≥8，R | SYN-R | SYN-S | ≥8，R | 1，S | ≤0.12，S | 8，R | ≤16，S | 8 |
| EF-2216 | ≤2，S | 2，S | ≤0.5，S | ≤0.25，S | 1，S | ≥16，R | ≥8，R | SYN-S | SYN-S | ≥8，R | 1，S | ≤0.12，S | 4，R | ≤16，S | 8 |
| EF-6166 | ≤2，S | 2，S | ≥8，R | ≥8，R | ≥8，R | ≥16，R | ≥8，R | SYN-S | SYN-R | ≥8，R | 1，S | ≤0.12，S | 4，R | ≤16，S | 16 |
| EF-5015 | ≤2，S | 1，S | ≥8，R | 4，R | ≥8，R | ≥16，R | ≥8，R | SYN-R | SYN-R | ≥8，R | 1，S | ≤0.12，S | 8，R | 64，I | 8 |
| EF-2021 | ≤2，S | 2，S | ≥8，R | 4，R | ≥8，R | ≥16，R | ≥8，R | SYN-S | SYN-R | ≥8，R | 2，S | ≤0.12，S | ≥16，R | ≤16，S | 16 |
| EF-8014 | ≤2，S | 1，S | ≥8，R | 4，R | ≥8，R | ≥16，R | ≥8，R | SYN-R | SYN-R | ≥8，R | 1，S | ≤0.12，S | 8，R | ≤16，S | 8 |
| EF-3186 | ≤2，S | 2，S | 2，I | 0.5，S | 2，S | ≥16，R | ≥8，R | SYN-S | SYN-S | ≥8，R | 1，S | ≤0.12，S | 4，R | ≤16，S | 8 |
| EF-1090 | ≤2，S | 2，S | ≥8，R | ≥8，R | ≥8，R | ≥16，R | ≥8，R | SYN-R | SYN-R | ≥8，R | 1，S | ≤0.12，S | 2，R | ≤16，S | 8 |

^a^Determined in our previous study(Hua et al., 2019).

AMP, ampicillin; PEN, penicillin G; CIP, ciprofloxacin; MOX moxifloxacin; LEV, levofloxacin; TET, tetracycline; ERY, erythromycin; HLSR, high-level streptomycin resistance; HLGR, high-level gentamicin resistance; CLI, clindamycin; VAN, vancomycin; TIG, tigecycline; DAF, quinupristin/dalofopine; FUR, nitrofurantoin; LZD, linezolid. R, resistant; S, susceptible,

Supplementary Table S2. Primers used for PCR

| Gene | primer sequence(5`-3`) | product size (bp) | reference |
| --- | --- | --- | --- |
| ***optrA*** | ATCAACTGTTCCCATTCA | 1395 | (Hua et al., 2019) |
|  | ATCAACTGTTCCCATTCA |  |  |
| ***tetL*** | TTGGTCCTATCTTCTACTCAT | 705 | This study |
|  | GCAATACCTGTTCCCTCT |  |  |
| ***tetM*** | GAAAAGGTACTAAACCAAATA | 501 |  |
|  | AGTAACGGTACTTAAATTGTTTAC |  |  |
| ***ermB*** | GAAAAGGTACTAAACCAAATA | 616 |  |
|  | AGTAACGGTACTTAAATTGTTTAC |  |  |
| ***lnuB*** | CTTATCTAATCGAGCAGTG | 450 |  |
|  | TACGGAACAGAGGTAGTTATT |  |  |
| ***fexA*** | TGGAGTTCCGATTTATGG | 977 |  |
|  | CACTTACTGCGGCGTTAT |  |  |
| ***rep*1** | TCGCTCAATCACTACCAAGC | 624 | (Jensen et al., 2010) |
|  | CTTGAACGAGTAAAGCCCTT |  |  |
| ***rep*2** | GAGAACCATCAAGGCGAAAT | 630 |  |
|  | ACCAGAATAAGCACTACGTACAATCT |  |  |
| ***rep*3** | CCTAATGTATATAATTTTGGTACATAT | 403 |  |
|  | ACATTTTCCTCAAAGAACAT |  |  |
| ***rep*4** | ACTATGTCGTTGAGTCTAATGACT | 430 |  |
|  | AGCAAGATAGAATATTTACTTTTAAGTTT |  |  |
| ***rep*5** | ATGTGTAATAAATTAAAAGAGCA | 637 |  |
|  | ATTGTCTTGATTTATCTATCTTG |  |  |
| ***rep*6** | ACGAATGAAAGATAAAGGAGTAG | 551 |  |
|  | TAAATTCTAGTTTGGCAATCTTA |  |  |
| ***rep*7** | AGACGTAATATGCGTRTTGA | 227 |  |
|  | CCAAAATAYTTYGTTTCTGG |  |  |
| ***rep*8** | TAGATACGACAAAAGAAGAATTACA | 394 |  |
|  | CCAATCATGTAATGTTACAACC |  |  |
| ***rep*9** | GCTCGATCARTTTTCAGAAG | 201 |  |
|  | CGCAAACATTTGTCWATTTCTT |  |  |
| ***rep*10** | TATAAAGGCTCTCAGAGGCT | 382 |  |
|  | CCAAATTCGAGTAAGAGGTA |  |  |
| ***rep*11** | TCTAGAATGCGTAAAAAGG | 500 |  |
|  | CCTTTGAAGATWGCRGTWAG |  |  |
| ***rep*12** | GAGCCTATAACAGAGTACACA | 470 |  |
|  | CAAATATAGGCTTTGTAGTTC |  |  |
| ***rep*13** | CAAATATAGGCTTTGTAGTTC | 402 |  |
|  | TACCAGAATAYTTAGCCATTTC |  |  |
| ***rep*14** | GAAAGYTTRGATAGYTTTGC | 164 |  |
|  | RTTTTGRCTTTCTTSYTTCA |  |  |
| ***rep*15** | CAGTAGAAGAAAATTATAAAGAAC | 327 |  |
|  | GTTATGGCTGGTTTTAATAAA |  |  |
| ***rep*16** | CAGGAAAACACTTCGTTTAT | 592 |  |
|  | CTTCTATATCACTATCATTGTCATT |  |  |
| ***rep*17** | TACTAACTGTTGGTAATTCGTTAAAT | 604 |  |
|  | ATCAAGGACTCAACCGTAATT |  |  |
| ***rep*18** | ACACCAGTCGAAATGAATTT | 462 |  |
|  | AGGAATATCAAGTAATTCATGAAAGT |  |  |
| ***rep*19** | GWGATCGCTTARAYTTATCTAT | 543 |  |
|  | YMTTGTTSTGGMAATTCTT |  |  |
| ***rep*-unique** | GTATTAACACACTGGACTC | 199 |  |
|  | TCAGTGTAGGCAATAACCC |  |  |
| ***prgA*** | GTATTTAAGGCTTGCTGACT | 797 | This study |
|  | AAAGGGCATAAAGGGATT |  |  |
| ***prgB*** | GACAGGGGATACAACAGAAC | 427 | (Donelli et al., 2004) |
|  | TGCGGCTAAACTACCAAC |  |  |
| ***prgC*** | TGTTCCTGCCGATTGATT | 462 | This study |
|  | GCATTCGCTGACGATTTA |  |  |

**Supplemental Table S3**. Genes encoded on plasmid pEF10748.

| CDS | pEF10748 location | Gene/protein size (bp/AA) | Direction | Predicted gene | Product | GenBank accession no. for the top hit | Identities  (%) |
| --- | --- | --- | --- | --- | --- | --- | --- |
| 1 | 75-695 | 621/206 | cw |  | Recombinase family protein [*Enterococcus faecalis*] | WP_013438829.1 | 100 |
| 2 | 712-996 | 285/94 | cw |  | Hypothetical protein [Firmicutes] | WP_002394798.1 | 100 |
| 3 | 998-1231 | 234/77 | cw |  | Hypothetical protein [Firmicutes] | WP_002394799.1 | 100 |
| 4 | 1391-1645 | 255/84 | ccw |  | Hypothetical protein | WP_002394800.1 | 100 |
| 5 | 1762-2430 | 669/222 | ccw |  | CPBP family intramembrane metalloprotease | WP_002403283.1 | 100 |
| 6 | 2466-2783 | 318/105 | ccw |  | Heavy metal-binding domain-containing protein | WP_002394802.1 | 100 |
| 7 | 3628-3927 | 300/99 | cw | *prgN* | Type III secretion system protein PrgN, partial [*Enterococcus faecalis*] | WP_122935380.1 | 83 |
| 8 | 4712-5254 | 543/180 | ccw |  | Hypothetical protein | WP_086321354.1 | 100 |
| 9 | 5264-6115 | 852/283 | ccw | *parA* | ParA family protein [*Enterococcus*] | WP_002362419.1 | 100 |
| 10 | 6778-7794 | 1017/338 | cw | *repA* | Replication protein RepA [*Enterococcus faecalis*] | WP_033593833.1 | 100 |
| 11 | 7940-9100 | 1149/382 | cw | *traB* | TraB/GumN family protein [*Enterococcus faecalis*] | WP_002394863.1 | 99 |
| 12 | 9155-10000 | 846/281 | ccw |  | Helix-turn-helix transcriptional regulator [*Enterococcus*] | WP_002394862.1 | 92 |
| 13 | 10828-11208 | 381/126 | cw | *prgR* | Pheromone-responsive regulatory protein R [*Enterococcus*] | WP_002394861.1 | 100 |
| 14 | 11208-11480 | 267/88 | cw | *prgS* | PrgS protein (plasmid) [*Enterococcus faecalis* T11] | EEU92067.1 | 99 |
| 15 | 11781-12086 | 306/101 | cw | *prgT* | Putative regulatory protein PrgT [*Enterococcus faecalis* OG1RF] | P0DH73.1 | 96 |
| 16 | 12097-14772 | 2676/891 | cw | *prgA* | SEC10/PgrA surface exclusion domain-containing protein [*Enterococcus faecalis*] | WP_086321355.1 | 99 |
| 17 | 14916-15251 | 336/111 | cw |  | Hypothetical protein [Bacteria] | WP_002360910.1 | 100 |
| 18 | 15541-19458 | 3918/1305 | cw | *prgB* | LPXTG-anchored aggregation substance PrgB/Asc10 [*Enterococcus faecalis*] | WP_086321356.1 | 100 |
| 19 | 19556-19876 | 321/106 | cw | *prgU* | pheromone response system RNA-binding regulator PrgU [*Enterococcus faecalis*] | WP_113826367.1 | 90 |
| 20 | 19898-20284 | 387/128 | cw |  | Hypothetical protein [*Enterococcus faecalis*] | WP_128704346.1 | 99 |
| 21 | 20352-20612 | 261/86 | cw |  | Hypothetical protein [*Enterococcus faecalis*] | WP_048942712.1 | 98 |
| 22 | 20623-21501 | 879/292 | cw |  | Hypothetical protein [*Enterococcus faecalis*] | WP_029888160.1 | 100 |
| 23 | 21521-22363 | 843/280 | cw | *prgC* | LPXTG cell wall anchor domain-containing protein [*Enterococcus faecalis*] | WP_086321358.1 | 100 |
| 24 | 22389-23660 | 1272/423 | cw | *chap* | CHAP domain-containing protein [*Enterococcus faecalis*] | WP_010773576.1 | 100 |
| 25 | 23663-24280 | 618/205 | cw |  | Hypothetical protein [*Enterococcus faecalis*] | WP_086321359.1 | 100 |
| 26 | 24312-24680 | 369/138 | cw |  | Hypothetical protein HMPREF9509_02626 [*Enterococcus faecalis* TX0411] | EFM66179.1 | 88 |
| 27 | 24680-24994 | 315/104 | cw |  | Orf13 [*Enterococcus faecalis* OG1X palsmid pAD1] | CAA65672.1 | 99 |
| 28 | 24987-26021 | 1035/344 | cw |  | Conjugal transfer protein [*Enterococcus faecalis*] | WP_002403100.1 | 100 |
| 29 | 26011-26286 | 276/91 | cw |  | Hypothetical protein [*Enterococcus faecalis*] | WP_010904443.1 | 98 |
| 30 | 26286-26675 | 390/129 | cw |  | Conjugal transfer protein [*Enterococcus faecalis*] | WP_011109607.1 | 97 |
| 31 | 26707-27189 | 483/160 | cw |  | Hypothetical protein [*Enterococcus*] | WP_002360790.1 | 100 |
| 32 | 27284-27742 | 459/152 | cw | *ssb* | Single-stranded DNA-binding protein [*Enterococcus faecalis*]a | WP_010822270.1 | 99 |
| 33 | 28051-30339 | 2289/762 | cw |  | Type VI secretion protein [*Enterococcus faecalis*] | OIU90382.1 | 90 |
| 34 | 30356-30700 | 345/114 | cw |  | Hypothetical protein [*Enterococcus faecalis*] | WP_010818139.1 | 100 |
| 35 | 30895-33057 | 2163/720 | cw |  | Hypothetical protein [*Enterococcus faecalis*] | WP_010822506.1 | 92 |
| 36 | 33119-35302 | 2184/727 | cw |  | TraM recognition domain-containing protein [*Enterococcus faecalis*] | WP_013438834.1 | 97 |
| 37 | 35304-35474 | 171/56 | ccw |  | Hypothetical protein [*Enterococcus*] | WP_002367755.1 | 100 |
| 38 | 35960-36610 | 651/216 | cw |  | ArsR family transcriptional regulator [*Enterococcus faecalis*] | WP_002377944.1 | 89 |
| 39 | 36616-37104 | 489/162 | cw |  | Hypothetical protein [*Enterococcus faecalis*] | WP_086321361.1 | 100 |
| 40 | 37330-37704 | 375/124 | cw |  | Hypothetical protein [*Enterococcus faecalis*] | WP_000108764.1 | 100 |
| 41 | 37711-37977 | 267/88 | cw |  | Hypothetical protein [*Enterococcus faecalis*] | WP_001245899.1 | 100 |
| 42 | 38139-38915 | 777/258 | cw |  | Hypothetical protein [Bacilli] | WP_000347505.1 | 100 |
| 43 | 38912-40246 | 1335/444 | cw | *pcfJ* | PcfJ [Bacilli] | WP_002372583.1 | 99 |
| 44 | 40386-40862 | 477/158 | cw | *ssb* | Single-stranded DNA-binding protein [Bacteria] | WP_000615486.1 | 100 |
| 45 | 41081-41227 | 147/48 | ccw |  | Putative holin-like toxin [*Enterococcus faecalis*] | WP_141435800.1 | 100 |
| 46 | 41799-41996 | 198/65 | cw |  | Hypothetical protein HMPREF9376_01254 [*Enterococcus faecalis* S613] | EFE19718.1 | 84 |
| 47 | 42193-43476 | 1284/427 | cw | *tnp* | ISL3 family transposases [*Enterococcus faecalis*] | AKA86804.1 | 100 |
| 48 | 43469-43690 | 222/73 | ccw |  | Hypothetical protein (plasmid) [*Enterococcus faecalis*] | AKA86805.1 | 100 |
| 49 | 43690-44250 | 561/186 | ccw |  | Hypothetical protein [*Enterococcus*] | WP_065813922.1 | 100 |
| 50 | 44452-45723 | 1272/423 | cw | *impB* | Type VI secretion protein ImpB [*Enterococcus faecalis*] | AMM74566.1 | 99 |
| 51 | 45747-46118 | 372/123 | cw |  | Hypothetical protein [*Enterococcus*] | WP_065813869.1 | 100 |
| 52 | 46359-46796 | 438/145 | ccw |  | Hypothetical protein (plasmid) [*Enterococcus faecalis*] | AKA86809.1 | 100 |
| 53 | 47503-47646 | 144/47 | cw |  | Hypothetical protein (plasmid) [*Enterococcus faecalis*] | AQM74931.1 | 100 |
| 54 | 47900-49327 | 1428/475 | cw | *fexA* | Chloramphenicol/florfenicol efflux MFS transporter FexA [*Enterococcus*] | WP_065813868.1 | 100 |
| 55 | 49505-49696 | 192/63 | cw |  | Hypothetical protein (plasmid) [Enterococcus faecalis] | AKA86812.1 | 100 |
| 56 | 49724-49909 | 186/61 | cw |  | Hypothetical protein (plasmid) [*Enterococcus faecalis*] | AKA86813.1 | 98 |
| 57 | 50063-51982 | 1920/639 | cw | *optrA* | ABC-F type ribosomal protection protein OptrA [Lactobacillales] | WP_063854496.1 | 97 |
| 58 | 52401-53081 | 681/226 | cw | IS1216 | IS6 family transposase (plasmid) [*Enterococcus faecalis*] | ARQ19070.1 | 97 |

CW, clockwise; CCW, counter clockwise.

Supplementary Table S4. Homology of plasmid pEF10748 with selected known plasmids in *E. faecalis.*

| Plasmid | Main overlap regions in pEF10748 | Overlap coverage (%) | Identity (%) | Bacterial strain | Main genes in homologous regions | GenBank accession nos. |
| --- | --- | --- | --- | --- | --- | --- |
| pKUB3006-1 | 1-41579 | 78 | 99.9 | *E. faecalis* KUB3006 | Sex pheromone response genes | AP018539.1 |
| pE211 | 17231-42033 | 74 | 98.5 | *E. faecalis* E211 | Sex pheromone response genes, *optrA* | MK425644.1 |
| pE508 | 10379-34039 | 67 | 96.9 | *E. faecalis* E508 | Sex pheromone response genes, *optrA* | MK425645.1 |
| pAD1 | 27284 -42033 | 29 | 99.1 | *E. faecalis* DS16 | Sex pheromone response genes , conjugation related gene | AH011360.2 |
| pCF10 | 15511-19881 | 22 | 96.0 | *E. faecalis* SF-7 | Sex pheromones response genes | NC_006827.2 |
| pMG2200 | 14825-19875 | 24 | 96.0 | *E. faecalis* NKH15 | Sex pheromone response genes | AB374546.1 |
| pTEF1 | 17231-26706 | 56 | 98.0 | *E. faecalis* V583 | Similar to pAD1 | AE016833.1 |
| pTEF2 | 15511-19881 | 20 | 96.0 | *E. faecalis* V583 | Similar to pCF10 but lacking *prgQ* | AE016831.1 |
| pXY17 | 42100-53135 | 20 | 99.9 | *E. faecalis* XY17 | Resistant genes *optrA* and *fexA* | KT862780.1 |
| p10-2-2 | 42312-53135 | 20 | 99.9 | *E. faecalis* 10-2-2 | Resistant genes *optrA* and *fexA* | KT862775.1 |
| p29462 | 42034 -53135 | 20 | 99.9 | *E. faecalis* 29462, | Resistant genes *optrA* and *fexA* | MH225419.1 |
| p1202 | 42034-53125 | 20 | 99.9 | *E. faecalis* 1202 | Resistant genes *optrA* and *fexA* | MH225414.1 |
| pE121 | 42034-53125 | 20 | 99.9 | *E. faecalis* E121 | Resistant genes *optrA* and *fexA* | KT862776.1 |

**
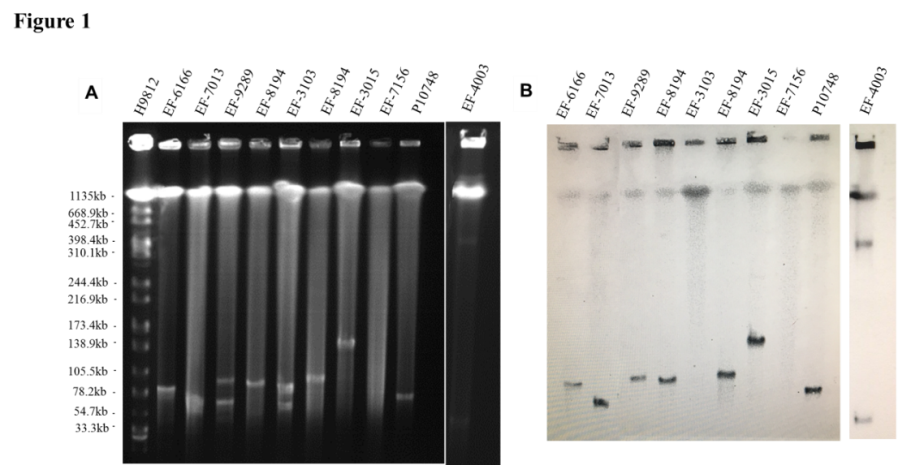

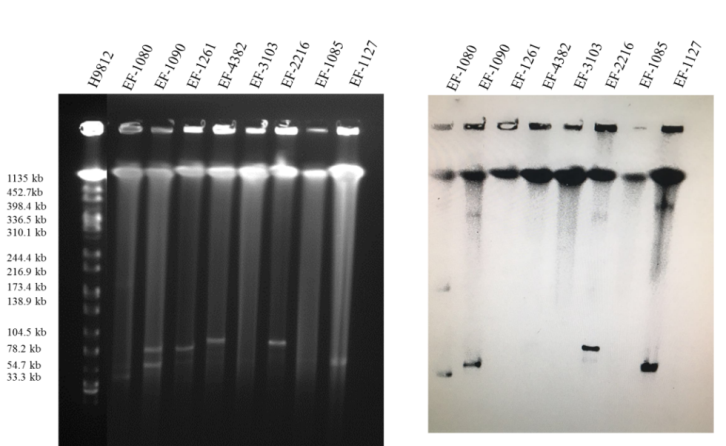

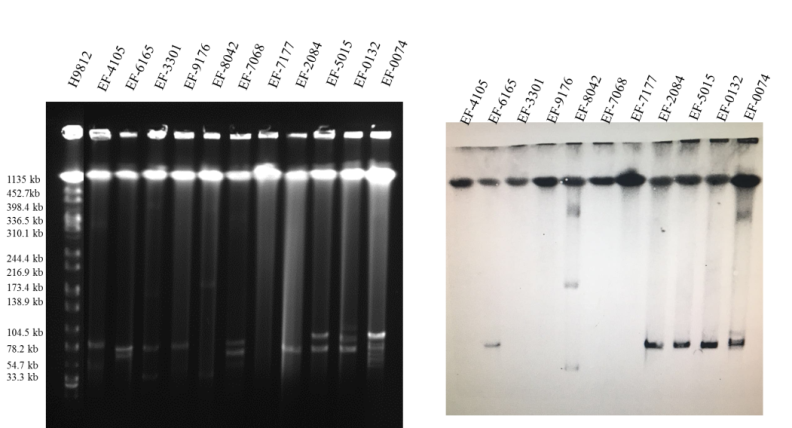

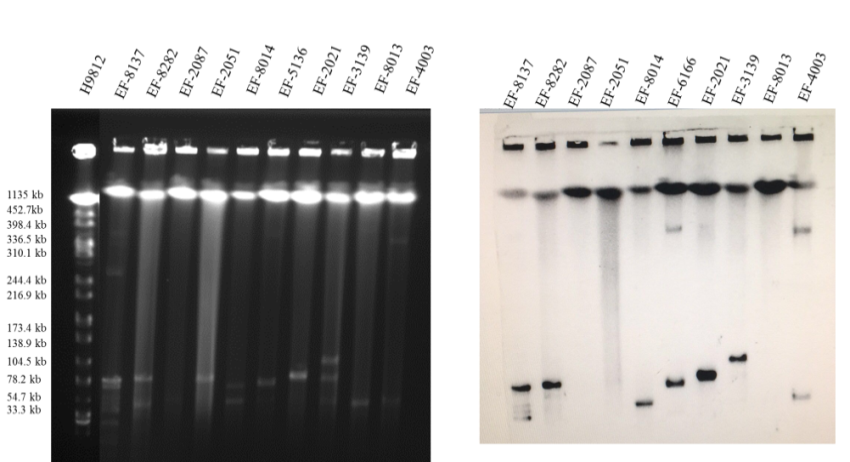

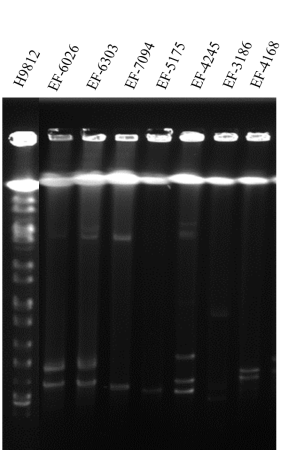
**

**
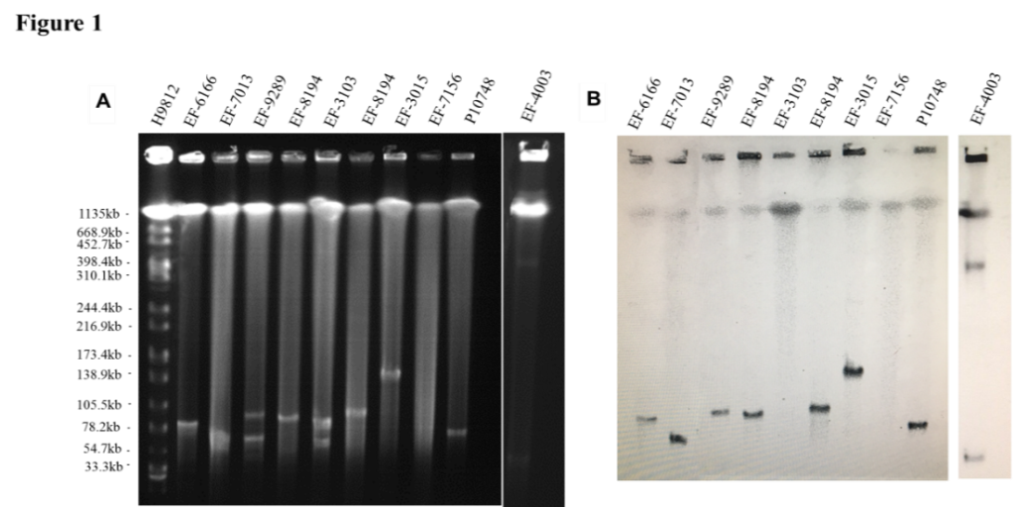

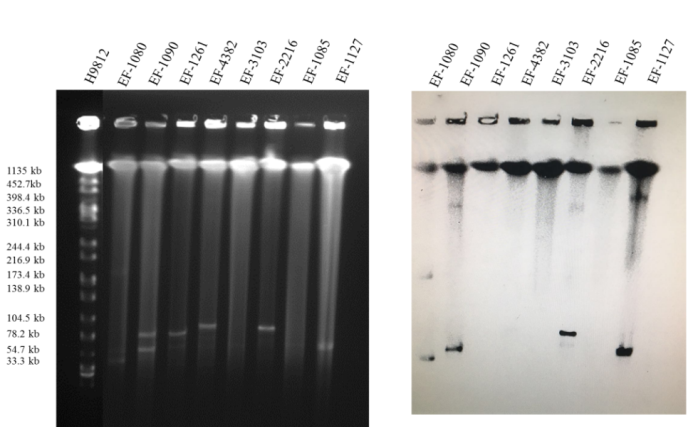

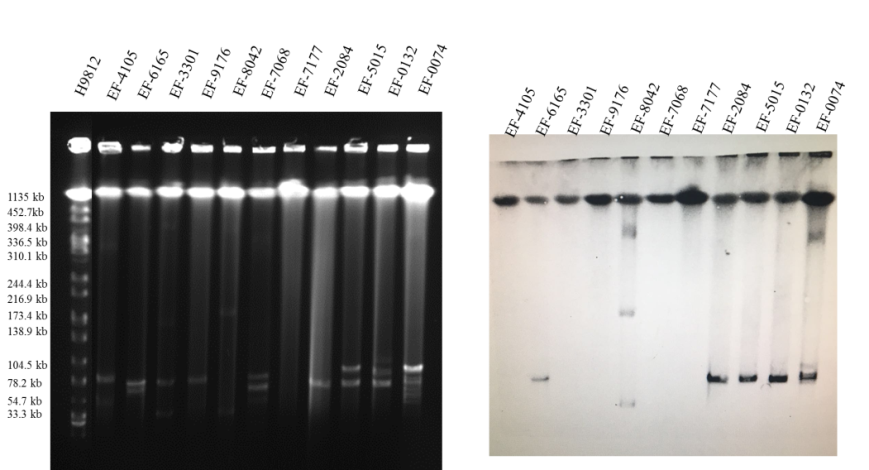

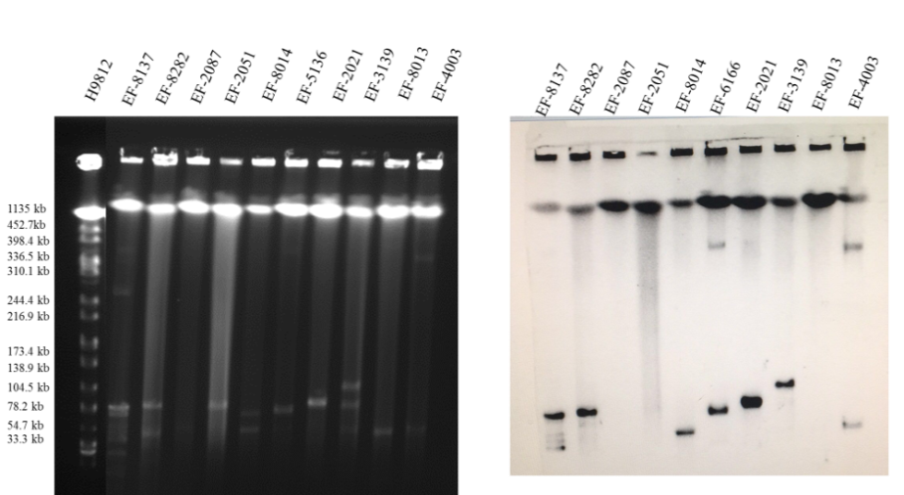

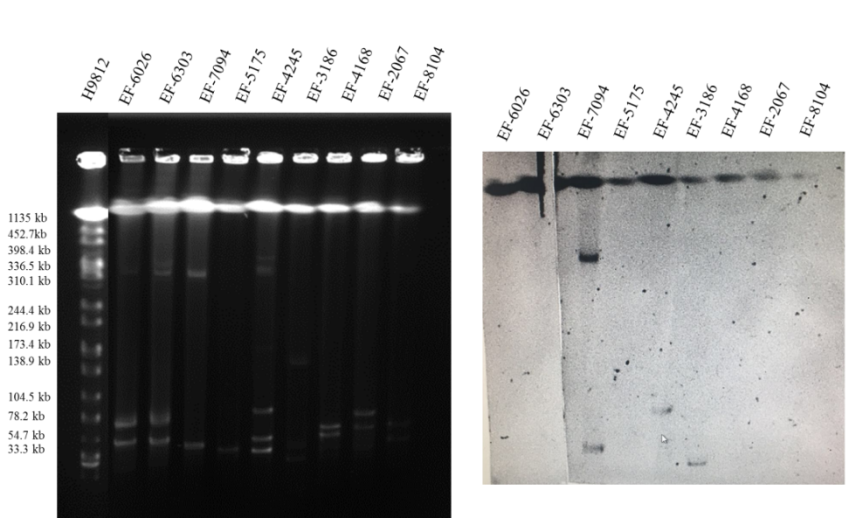
**

*

*

*

**Supplement Figure S1**. Determination of the location of *optrA* in 44 linezolid-resistant *E. faecalis* isolates. (A) Representative results of S1 nuclease-pulsed-field gel electrophoresis analysis. Gels were stained with ethidium bromide. In each gel, the first lane contained *XbaI*-digested chromosome of *Salmonella* H9812 as a DNA size marker while each other lane contained individual *E. faecalis* isolate as listed in Table 1, with its code labeled on the top. (B) Representative results of Southern hybridization showing the location of *optrA* in plasmids or chromosomes. Labels above each lane represent sample codes for individual *E. faecalis* isolates as listed in Table 1. Isolates potentially containing two or more plasmids are indicated by *. Isolates EF-6166 and EF-8194 are shown twice in multiple lanes and gels.
